# Estimating Prevalence and Number of People With Chronic Hepatitis B: A Multiplier Method Based on Public Health Surveillance Data in UK (2015–2021)

**DOI:** 10.1111/jvh.14019

**Published:** 2024-10-16

**Authors:** Ruth Simmons, Ross Harris, Aaron G. Lim, David Leeman, Mary E. Ramsay, Matthew Hickman, Sema Mandal

**Affiliations:** ^1^ Blood Safety, Hepatitis, Sexually Transmitted Infections (STIs) and HIV Division UK Health Security Agency London UK; ^2^ NIHR HPRU in Behavioural Science and Evaluation University of Bristol Bristol UK; ^3^ Population Health Sciences, Bristol Medical School University of Bristol Bristol UK

**Keywords:** ethnic group, HBV, Hepatitis B virus, prevalence estimation

## Abstract

Estimates of chronic hepatitis B virus (HBV) prevalence and critically the amount of infection that is undiagnosed or unlinked to care are uncertain—even in countries like UK where vertical transmission and overall prevalence are very low. In the absence of country of birth data, we aim to estimate HBV prevalence through combining public health surveillance data on antenatally screened women by ethnic group and multipliers generated from non‐antenatally screened populations by ethnic group with English population denominators. Of 714,287 women aged 16–49 years with ethnic group data tested as part of antenatal care between 2015 and 2021, 4174 (0.6%) were HBsAg‐positive; 94% in people of ethnic groups other than White British. Of 1,447,467 people tested for HBsAg with ethnic group data from other testing sources (primary and secondary care excluding occupational health and renal services), 27,628 (1.9%) were HBsAg‐positive; 87% in people of ethnic groups other than White British. We estimate that the overall number and prevalence of people with chronic hepatitis B in England is 268,767 (95% CI: 227,896–314,044) and 0.58% (95% CI: 0.50–0.68). Approximately two‐thirds were male, one‐third female, and 68% were aged under 50. We estimate that over 83% of HBV infections are in people of ethnic groups other than White British, with 23% in people from Black ethnic groups, 21% from other White ethnic groups and 19% in Asian ethnic groups. These estimates are the first step towards establishing whether England can meet World Health Organisation targets to eliminate HBV as a public health problem—using methods that can also be used by other countries.

## 1 Introduction

Globally, Hepatitis B virus (HBV) infection is common with approximately 4% (over 300 million) of people infected and is the leading cause of end‐stage liver disease and liver cancer [1, 2], exceeding mortality estimates for HIV and hepatitis C virus (HCV) [3, 4, 5]. HBV has been recognised as a major public health problem in the 2016 WHO Global Health Sector Strategy for the Elimination of Viral Hepatitis [6]. In the UK, compared to many other countries, the prevalence of chronic hepatitis B infection is very low (< 1%) but highly focussed, with the majority of those diagnosed having acquired their infection overseas in endemic countries and migrated to the UK [7]. In the UK, universal childhood HBV vaccination was added to the childhood schedule (with hexavalent vaccine) from 2017 [8]. There is very little vertical transmission of HBV given high uptake of universal antenatal screening (> 99%) combined with high coverage of selective immunisation of infants born to pregnant women with HBV‐identified through screening (> 96% first (birth) dose coverage), and subsequent doses in the hexavalent programme [8].

However, HBV is still a neglected health problem [9] with a lack of evidence on the size of the population and burden, and uncertainty over the proportion of the population with chronic hepatitis B that have been diagnosed and linked to treatment and care, evidence that is essential for measuring whether World Health Organisation (WHO) HBV elimination targets have been met, including that ≥ 90% of people with HBV are diagnosed, and ≥ 80% of people diagnosed with HBV and eligible for treatment are treated [6].

There are no recent robust England estimates for chronic hepatitis B burden. Owing to the often asymptomatic nature of both acute infection, which is age‐dependent, and chronic infection until liver complications arise decades after acquisition of infection, laboratory diagnoses alone do not equate to prevalence as they do not capture untested and hence undiagnosed people, noting that testing in England is mainly risk or symptoms based rather than through population‐level screening programmes. In 2013, the National Institute for Health and Care Excellence (NICE) estimated that there may be as many 500,000 chronically infected individuals in the UK [10], whereas the department of health estimated 180,000 in 2002 [7, 11]. In this study, we use a multiplier approach [12] applied to public health surveillance data from laboratories to estimate the number of people living with chronic hepatitis B in England.

## 2 Methods

We estimate the prevalence and number of people living with chronic hepatitis B through combining HBV laboratory test data in antenatally screened women, multipliers derived from non‐antenatally screened population in ethnic groups and population estimates stratified by age and ethnic group.

### 2.1 Laboratory Data

Sentinel Surveillance of Bloodborne Virus Testing in England (SSBBV) is a unique surveillance system that captures all blood‐borne virus tests regardless of the result for tests conducted at any one of 23 sentinel laboratories across England. Participating laboratories are estimated to cover approximately 40% of the English population for primary and reference HBV testing and are broadly representative of most laboratories providing blood borne virus testing. Data collection methods for SSBBV have been described previously [13]. In summary, demographic and testing data for all individuals tested for hepatitis B surface antigen (HBsAg) and/or immunoglobulin M antibody against core antigen (anti‐HBc IgM), the latter an indication of acute infection, are extracted from participating laboratory information systems. Individuals are de‐duplicated, and test results for each individual are linked over time using a combination of soundex code (a coding based on the person's surname), first initial, date of birth and NHS number.

Two files were extracted from SSBBV; results from all women aged 16–49 years old tested as part of routine antenatal screening between 2015 and 2021, and individuals ≥ 16 years identified as non‐antenatal testing, with a HBsAg test during the same time period. The test request location and clinical details accompanying the test request were used to distinguish women assumed to be tested for HBsAg as part of routine antenatal screening from those tested in other settings and for other reasons. For those with multiple tests in the study period, the first test was used in analyses.

### 2.2 Assignment of Ethnicity

In the absence of country of birth data, we assigned ethnic group using data collected in NHS England Hospital Episode Statistics (HES). Individuals reported to SSBBV were linked to the hospital inpatient, outpatient and emergency department datasets using NHS number. Ethnicity was categorised into six groups:

- White British;
- White other including White Irish;
- Asian including Chinese, Bangladeshi (Asian or Asian British), Pakistani (Asian or Asian British), Indian (Asian or Asian British), any other Asian background;
- Black including African (Black or Black British), Caribbean (Black or Black British), any other Black background;
- Mixed including any other Mixed background, White and Asian (Mixed), White and Black African (Mixed), White and Black Caribbean (Mixed);
- Other including any other ethnic group.

### 2.3 Exclusions

Individuals with an anti‐HBc IgM positive result within 1 month of being HBsAg positive and/or had a negative HBsAg within 6 months were excluded as indicated an acute infection, which, in adults generally results in virus clearance rather than chronic infection. Acute infections were also identified by linking to the reports of an acute infection reported by local teams using NHS number. Those tested through sources investigating sub‐groups of the population also were excluded (including occupational health and renal services). Finally, individuals who had conflicting ethnic groups through linkage were excluded, as the true ethnicity of the individual could not be established.

### 2.4 Population Estimates

Population estimates were based on the 2021 census published by the Office for National Statistics. The dataset used was broken down by age, sex and ethnic group for all residents [14].

### 2.5 Data Processing and Analysis

SQL was used for data storage and data linkage. Statistical analyses were performed using STATA version 15 (Stata Corp., College Station, Tx, USA).

### 2.6 Prevalence Estimates

In the method proposed by Schnier et al. [12] we assume (a) that HBV positivity in women of childbearing age by ethnic group is represented by HBV antenatal screening as it has > 99% uptake [15]; and (b) the difference in prevalence between men and women and by age is represented by the ratio of HBV positivity in non‐antenatal testing. In the latter, we are not assuming that the prevalence of non‐antenatal testing is representative but relative differences by age and gender are represented by these data. Algebraically, prevalence for each ethnicity *e*, age group *a* and sex *s* is therefore obtained via

$$\pi_{e,a,s}=\pi_{e,1,1}\beta_{e,a,s}$$

where for ethnicity *e*: $\pi_{e,1,1}$ is the prevalence in the baseline age and sex group (namely, women aged 16–29), and $\beta_{e,a,s}$ is the age‐ and sex‐specific RR for age group *a* (which is 1 if *a* = 1), and sex group (which is 1 if *s* = 1, and represents the RR for males vs. females if *s* = 2). An interaction was identified between age and sex for all ethnicities, as a result a single age‐sex grouping variable was created (female 16–29 years, female 30–49 years, female ≥ 50 years, male 16–29 years, male 30–49 years, male ≥ 50 years). Confidence intervals (CI) are derived using bootstrapping techniques. The log odds ratios and their standard errors from the logistic regression model above were used to simulate 1000 log odds ratios from the normal distribution $N\left(\log\left(\beta_{e,a,s}\right),\sigma_{e,a,s}\right)$. These were combined with 1000 bootstrap samples of the binomial antenatal data to produce a distribution of 1000 simulated values of each $\pi_{e,a,s}$. The resulting distributions were summarised via the 2.5th and 97.5 percentiles to construct 95% confidence intervals (see, e.g., [16]).

Using population estimates of age, sex and ethnicity for England, we extrapolated the number of people with chronic hepatitis B using the estimated prevalence estimates.

### 2.7 Ethics

UK Health Security Agency has approval from the Secretary of State for Health and Social Care under ‘section 251’ of the National Health Service Act 2006 and the associated Health Service (Control of Patient Information) Regulations 2002, to collect and process confidential patient information without consent for diagnosing, recognising trends, controlling and preventing, and monitoring and managing communicable diseases and other risks to public health. Therefore, no research or ethics review is required.

## 3 Results

### 3.1 HBV Positivity in Females Aged 16–49 Years by Ethnicity

Among the 793,463 women aged 16–49 years who were tested as part of antenatal care between 2015 and 2021 and reported by laboratories participating in SSBBV, 4560 (0.6%) were HBsAg positive. Data on ethnicity were available on 714,287 (90.0%) and 4174 (91.5%) of women antenatally tested and those who were HBsAg‐positive, respectively. Table 1 shows HBsAg positivity among those tested through antenatal screening. The majority of those who were tested were of White British (59.5%), followed by White other (including White Irish) (14.4%) and Asian (12.8%) ethnic groups. Among those who tested HBsAg positive in antenatal test settings, 94% were in ethnic groups other than White British, with 30% in Black ethnic groups and 23% in White other ethnic groups. HBV positivity was highest in people of other ethnic groups (3.0%), followed by those of Black ethnic groups (2.5%) (Table 1).

**TABLE 1:** Number of women aged 16–49 years tested for HBsAg as part of routine antenatal screening and test positivity, by ethnicity and age group between 2015 and 2021, reported by sentinel surveillance laboratories in England.

	HBsAg tested	HBsAg positive	Positivity (%)
Total	793,463	4560	0.57 (0.56–0.59)
Total with ethnicity data	714,287	4174	0.58 (0.57–0.60)
By ethnic group			
White British			
16–29	203,942	125	0.06 (0.05–0.07)
30–49	221,308	152	0.07 (0.06–0.08)
Both age groups	425,250	277	0.07 (0.05–0.07)
White other			
16–29	40,715	453	1.11 (1.02–1.20)
30–49	62,212	577	0.93 (0.86–0.99)
Both age groups	102,927	1030	1.00 (0.87–1.00)
Black			
16–29	23,123	444	1.92 (1.77–2.07)
30–49	30,720	915	2.98 (2.83–3.15)
Both age groups	53,843	1359	2.52 (2.37–2.67)
Asian			
16–29	40,478	263	0.65 (0.59–0.72)
30–49	50,936	443	0.87 (0.81–0.93)
Both age groups	91,414	706	0.77 (0.67–0.79)
Other			
16–29	8784	261	2.97 (2.67–3.27)
30–49	12,944	391	3.01 (2.80–3.27)
Both age groups	21,728	652	3.00 (2.61–3.10)
Mixed			
16–29	9180	46	0.50 (0.38–0.62)
30–49	9945	104	1.04 (0.89–1.21)
Both age groups	19,125	150	0.78 (0.60–0.88)
Region			
East Midlands	104,689	257	0.25 (0.22–0.28)
East of England	38,940	109	0.28 (0.23–0.34)
London	182,346	1842	1.01 (0.97–1.06)
North East	13,754	134	0.97 (0.82–1.15)
North West	138,292	689	0.50 (0.46–0.54)
South East	98,907	296	0.30 (0.27–0.34)
South West	85,056	354	0.42 (0.38–0.46)
West Midlands	54,623	387	0.71 (0.64–0.78)
Yorkshire and the Humber	76,648	445	0.58 (0.53–0.64)

### 3.2 HBV Positivity in Individuals Tested Through Non‐antenatal Settings

Table 2 shows non‐antenatal testing overall and by ethnic group, age and sex. Between 2015 and 2021, 2,207,126 individuals 16 years and over were tested in non‐antenatal settings, of which 38,195 (1.7%) were HBsAg‐positive (Table 2).

**TABLE 2:** Number of people tested for HBsAg (excluding women tested in antenatal screening) and test positivity, by ethnicity and age group between 2015 and 2021, reported by sentinel surveillance laboratories in England.

	HBsAg tested	HBsAg positive	Positivity (%)
Total	2,207,126	38,195	1.73
Sex			
Male	1,143,647	22,719	1.99
Female	1,037,059	14,693	
Not reported	26,420	783	2.96
Age group			
16–29	577,626	7200	1.25
30–49	978,350	22,156	2.26
≥ 50	642,377	8668	1.35
Not reported	8773	171	1.95
Setting of test			
Primary care	1,281,200	17,783	1.39
Secondary care	917,629	20,134	2.19
Not reported	8297	278	3.35
Region			
London	853,370	19,176	2.25
Outside London	1,347,118	18,749	1.39
Not reported	6638	270	4.07
Ethnicity			
White British	874,041	3567	0.41
White other (Inc. Irish)	178,869	4964	2.78
Asian	170,412	5000	2.93
Black	142,890	8363	5.85
Mixed	32,907	902	2.74
Any other	48,348	4832	9.99
Not reported	759,659	10,567	1.39

Ethnic group data were available for 1,447,467 (65.6%) individuals, with the majority categorised as White British (60.4%), followed by White other (including White Irish) (12.4%) and Asian ethnic groups (11.8%). Among those tested in non‐antenatal services where reported, 52.4% were female, 44.5% were aged 30–49 years, and 58.3% were tested in primary care services. HBsAg test positivity was higher in males than females (2.0% vs. 1.4%), higher in those aged 30–49 years (2.3%), higher among those testing in secondary care compared with primary care (2.2 vs. 1.4), and among those tested in London compared to outside London (2.3% vs. 1.4%). In non‐antenatal test settings, 87.1% of positive HBsAg tests were in ethnic groups other than White British, with 30.3% positive HBsAg tests in people in Black ethnic groups and approximately 17%–18% in people of White other, Asian, and other ethnic groups (Table 2). Test positivity was 1.9% (27,628) for those where ethnicity was reported. HBsAg positivity rates were highest among those of any other ethnicity (10.0%), followed by those of black ethnicity (5.9%), whereas individuals of White British ethnicity had the lowest HBsAg positivity rate at 0.4%.

People of White British, Black, Asian or other/mixed ethnicity regardless of sex, aged 30–49 years and ≥ 50 years were more likely to test positive for HBsAg compared with those aged 16–29 years (Table 3). Among people of White other ethnicity, compared to those aged 16–29 years, individuals aged 30–49 years were more likely to test positive, whereas those aged ≥ 50 years were less likely. We ran sensitivity analysis removing setting of test from the model and running separate models for primary and secondary care and identified little variation between the models.

**TABLE 3:** Generalised linear model adjusted relative risks for people tested for HBsAg (excluding women tested in antenatal screening) and test positivity, by ethnicity and age group between 2015 and 2021, reported by sentinel surveillance laboratories in England.

	HBsAg tested	HBsAg positive	Positivity (%)	Adjusted relative risk (95% CI)
Total with complete data	1,443,873	27,164	1.89	—
Female				
16–29	186,521	2196	1.18	1
30–49	341,834	7021	2.05	1.76 (1.69–1.83)
≥ 50	229,419	2624	1.14	0.96 (0.91–1.01)
Male				
16–29	127,414	2229	1.75	1.59 (1.52–1.67)
30–49	284,486	8762	3.08	2.64 (2.53–2.74)
≥ 50	264,199	4332	1.64	1.38 (1.32–1.44)
Test setting				
Primary	691,808	10,049	1.45	1
Secondary	750,170	17,499	2.33	1.73 (1.69–1.76)
By ethnicity group				
White British	863,541	3494	0.40	—
Female				
16–29	99,220	179	0.18	1
30–49	171,743	432	0.25	1.38 (1.16–1.65)
≥ 50	163,232	446	0.27	1.49 (1.25–1.77)
Male				
16–29	73,214	280	0.38	2.16 (1.79–2.61)
30–49	162,061	1079	0.67	3.72 (3.18–4.36)
≥ 50	194,071	1078	0.56	3.03 (2.58–3.55)
Test setting				
Primary	396,269	1533	0.39	1
Secondary	467,272	1961	0.42	1.13 (1.06–1.21)
White other (including White Irish)	177,042	4880	2.76	—
Female				
16–29	28,120	533	1.90	1
30–49	60,730	1412	2.33	1.17 (1.06–1.29)
≥ 50	17,881	330	1.85	0.93 (0.81–1.07)
Male				
16–29	12,514	372	2.97	1.73 (1.51–1.97)
30–49	37,962	1722	4.54	2.44 (2.21–2.68)
≥ 50	19,835	511	2.58	1.28 (1.13–1.44)
Test setting				
Primary	91,597	1875	2.05	1
Secondary	85,445	3005	3.52	1.83 (1.73–1.94)
Black	140,869	8204	5.82	—
Female				
16–29	20,163	627	3.11	1
30–49	38,506	2289	5.94	1.87 (1.71–2.03)
≥ 50	20,796	700	3.37	1.05 (0.95–1.17)
Male				
16–29	14,616	657	4.48	1.60 (1.43–1.78)
30–49	26,231	2733	10.42	3.50 (3.21–3.80)
≥ 50	20,557	1200	5.84	1.83 (1.67–2.01)
Test setting				
Primary	74,655	3179	4.26	1
Secondary	66,214	5025	7.59	1.86 (1.78–1.95)
Asian	167,190	4874	2.92	—
Female				
16–29	24,942	389	1.56	1
30–49	47,418	1263	2.66	1.68 (1.50–1.88)
≥ 50	18,807	482	2.56	1.62 (1.42–1.85)
Male				
16–29	16,309	491	3.01	2.30 (2.02–2.63)
30–49	38,961	1487	3.82	2.70 (2.41–3.01)
≥ 50	20,753	762	3.67	2.32 (2.06–2.62)
Test setting				
Primary	78,576	1401	1.78	1
Secondary	88,614	3473	3.92	2.34 (2.20–2.49)
Other	47,620	4749	9.97	—
Female				
16–29	6971	394	5.65	1
30–49	13,486	1387	10.28	1.62 (1.45–1.80)
≥ 50	4890	573	11.72	1.89 (1.67–2.13)
Male				
16–29	5556	339	6.10	1.21 (1.05–1.39)
30–49	11,424	1397	12.23	2.14 (1.92–2.38)
≥ 50	5293	659	12.45	1.97 (1.75–2.22)
Test setting				
Primary	26,932	1594	5.92	1
Secondary	20,688	3155	15.25	2.50 (2.36–2.65)
Mixed	32,459	890	2.74	—
Female				
16–29	6613	69	1.04	1
30–49	9103	220	2.42	2.21 (1.69–2.89)
≥ 50	2790	85	3.05	2.76 (2.02–3.78)
Male				
16–29	4705	87	1.85	2.06 (1.50–2.82)
30–49	6410	319	4.98	5.03 (3.89–6.51)
≥ 50	2838	110	3.88	3.50 (2.60–4.72)
Test setting				
Primary	17,513	317	1.81	1
Secondary	14,946	573	3.83	2.17 (1.90–2.49)

### 3.3 HBV Prevalence Estimates

Table 4 shows HBV estimates aggregated by sex, age, ethnicity, and region (London, outside London) in England. Using population estimates from the 2021 census broken down by age, sex and ethnicity for England, we estimate the number of people with chronic hepatitis B in England at 268,767 (227,896–314,044), equivalent to a prevalence estimate of 0.58% (95% CI: 0.50–0.68) (Table 4). Chronic hepatitis B prevalence was higher in London 1.52% (95% CI 1.17–1.97) than outside London 0.39% (95% CI 0.32–0.48).

**TABLE 4:** Estimated HBsAg prevalence and number of people with chronic hepatitis B.

	Estimated number of individuals with chronic hepatitis B	Estimated HBsAg prevalence (%), 95% CI
Total	268,767 (227,896–314,044)	0.58 (0.50–0.68)
Sex		
Female	96,444 (85,086–108,782)	0.41 (0.36–0.46)
Male	172,323 (142,810–205,262)	0.77 (0.64–0.92)
Age group		
16–29	54,781 (46,886–64,492)	0.56 (0.48–0.67)
30–49	128,695 (111,587–147,529)	0.86 (0.75–0.99)
≥ 50	85,292 (69,423–102,023)	0.40 (0.32–0.48)
Ethnicity group		
White British	45,892 (35,542–56,275)	0.13 (0.10–0.16)
White other	56,076 (49,131–63,501)	1.55 (1.36–1.76)
Black ethnic groups	60,649 (54,025–67,826)	3.38 (3.01–3.78)
Asian ethnic groups	50,454 (43,407–58,844)	1.22 (1.05–1.42)
Other ethnic groups	43,765 (37,563–50,917)	4.60 (3.95–5.36)
Mixed ethnic groups	11,932 (8228–16,680)	1.27 (0.88–1.78)
Region		
London	108,216 (83,316–140,120)	1.52 (1.17–1.97)
Outside London	150,894 (122,726–185,155)	0.39 (0.32–0.48)
By ethnic group		
White British		
Female		
16–29	1985 (1716–2267)	0.06 (0.05–0.07)
30–49	3389 (2971–3881)	0.07 (0.06–0.08)
≥ 50	8583 (6676–10,491)	0.09 (0.07–0.11)
Male		
16–29	4307 (3313–5632)	0.13 (0.10–0.17)
30–49	11,126 (8708–14,029)	0.23 (0.18–0.29)
≥ 50	16,501 (12,159–19,975)	0.19 (0.14–0.23)
White other		
Female		
16–29	4972 (4585–5362)	1.11 (1.02–1.20)
30–49	8875 (8252–9491)	0.93 (0.86–0.99)
≥ 50	5651 (4781–6574)	1.04 (0.88–1.21)
Male		
16–29	7198 (6169–8601)	1.90 (1.63–2.28)
30–49	23,222 (20,097–26,275)	2.71 (2.34–3.06)
≥ 50	6158 (5247–7199)	1.42 (1.21–1.66)
Black		
Female		
16–29	4965 (4580–5363)	1.92 (1.77–2.07)
30–49	11,349 (10,760–11,980)	2.98 (2.83–3.15)
≥ 50	6535 (5666–7468)	2.03 (1.76–2.32)
Male		
16–29	7673 (6742‐8880)	3.05 (2.68–3.53)
30–49	20,584 (17,930–23,286)	6.70 (5.84–7.58)
≥ 50	9544 (8347–10,849)	3.51 (3.07–3.99)
Asian		
Female		
16–29	3703 (3350–4092)	0.65 (0.59–0.72)
30–49	8455 (7830–9088)	0.87 (0.81–0.93)
≥ 50	6158 (5103–7332)	1.05 (0.87–1.25)
Male		
16–29	8510 (7075–10,119)	1.49 (1.24–1.77)
30–49	15,525 (13,206–18,470)	1.74 (1.48–2.07)
≥ 50	8102 (6843–9744)	1.50 (1.27–1.80)
Other		
Female		
16–29	3694 (3324–4068)	2.97 (2.67–3.27)
30–49	6464 (6017–7014)	3.01 (2.80–3.27)
≥ 50	7347 (6169–8762)	5.61 (4.71–6.69)
Male		
16–29	4666 (3914‐5722)	3.58 (3.00–4.39)
30–49	13,604 (11,465–15,850)	6.36 (5.36–7.41)
≥ 50	7990 (6674–9501)	5.87 (4.90–6.98)
Mixed		
Female		
16–29	1046 (797–1303)	0.50 (0.38–0.62)
30–49	1956 (1664–2267)	1.04 (0.89–1.21)
≥ 50	1317 (846–1980)	1.37 (0.88–2.06)
Male		
16–29	2062 (1321–3083)	1.03 (0.66–1.54)
30–49	4146 (2687–5899)	2.53 (1.64–3.60)
≥ 50	1405 (913–2149)	1.74 (1.13–2.66)

Among people with chronic hepatitis B, approximately two‐thirds (64%) were male and one‐third female (36%), with 68% aged under 50. We estimate that 83% of HBV infections are in people from ethnic groups other than White British, with 23% in people from Black ethnic groups, 21% from other White ethnic groups, 19% in Asian ethnic groups and 16% in other ethnic groups. HBV prevalence for men was highest among Black ethnic groups aged 30–49 years at 6.70% (95% CI 5.84–7.58) followed by those of other ethnic groups aged 30–49 years at 6.36% (95% CI 5.36–7.41). Among women, HBV prevalence was highest among by those of other ethnic groups aged ≥ 50 years at 5.61% (95% CI 4.71–6.69) followed by other ethnic groups aged 30–49 years 3.01% (95% CI 2.80–3.27). For both men and women, the lowest prevalence was among those of white British ethnicity regardless of age.

## 4 Discussion

### 4.1 Main Findings

Based on public health surveillance data (between years 2015–2021) from antenatal and non‐antenatal testing sources we estimate that prevalence of chronic hepatitis B was 0.58% (95% CI 0.50–0.68) or 268,767 individuals (228,000–314,000). Over 85% of positive HBsAg tests in both antenatal and non‐antenatal sources and an estimated 83% of the total number of people were from ethnic groups other than White British. We also estimated that two‐thirds of the people with chronic HBV were male, one‐third female, and approximately 70% were aged under 50 years. Prevalence of chronic HBV in London was nearly four times higher than outside London (1.52% vs. 0.39%).

### 4.2 Other Evidence

The baseline of the model uses data on women tested antenatally whose testing results were also reported by laboratories participating in SSBBV. These data are from a subset of all women screened for HBV in the NHS infectious Diseases in Pregnancy Screening (IDPS) programme. Compared to all women screened for HBV in IDPS, test positivity in testing was slightly higher: 1.14 for London and 0.39 for outside London in SSBBV for 2020, versus 0.65% in London and 0.26% outside London in IDPS programme data for the financial year 2020/21 [17]. These prevalence estimates are similar to those reported by Hahne et al. of 0.45% in England and Wales [7], and higher than previous estimates using surveillance data [18] due to changes to the census data related to the distribution of the underlying populations. However, our estimates are lower than the Global Burden of Disease estimates of approximately 0.7% [19]. Further work is needed to refine the prevalence model through statistical models using a similar method to that used for hepatitis C and HIV [20, 21, 22]. Gaps in data needed for statistical modelling should be identified and addressed using cross‐sectional data such as seroprevalence studies, with efforts to ensure the data are representative of the heterogeneous populations affected by HBV in England.

Compared to people of White British ethnicity, HBV prevalence estimates were higher in all other ethnic groups. The highest prevalence estimate were in those of Black ethnicity and Other ethnicity, both above the 2% threshold at which the WHO testing guidance suggest scale‐up of testing, diversifying approaches to reach those unaware of their infection [23]. Estimates for London are over three times higher than for outside London, with estimates similar to the 1.1% test positivity (including those with a known infection) observed in five London NHS trusts offering opt‐out blood‐borne virus (BBV) testing in emergency departments [24].

### 4.3 Strengths and Limitations

There are several limitations. First, the data used are derived from a sentinel network of laboratories, where, although covering approximately 40% of England's GP population, coverage varies geographically. This may introduce bias if sites missing in public health surveillance and more recent data have different ethnic groups, rates of testing and rates of HBV. Nonetheless, we believe the potential for biases from this limitation is small as the sample sizes are large, a prominent diagnostic laboratory in each area is included within SSBBV and although risk‐based testing rather than screening may occur in other settings, antenatal screening has almost 100% uptake. Second, the antenatal data for the seven year period 2015–2021 used as the baseline is a subset of all antenatal screening tests (793,463 women/~4,550,000 pregnancies) and so may not be fully representative. To explore how representative this baseline was, age, geographical distribution and ethnicity of screening results in SSBBV were compared to all live births [25]. Live birth data was used as demographic data is not published for the screening programme but with > 99% uptake it will be comparable with published live birth data. The age distribution among the subset was comparable with all births, however, there was some variation by region, with the West Midlands and the East of England underrepresented in SSBBV, and the East Midlands overrepresented. Due to the regional variability in SSBBV we present the prevalence estimates for London and outside London only and further work will be required to present additional regional breakdowns. While mothers ethnicity is not available in the live births data, the babies ethnicity is [26] and using this as a proxy for the mothers ethnicity the distribution was comparable, apart from those of mixed ethnicity which was lower among mothers in SSBBV compared to all babies, however, mixed ethnicity is difficult to compare as the babies ethnicity will represent the ethnicity of both parents. We also know that the positivity in our sample is slightly higher than the overall positivity reported by IDPS, and work is underway to include all women antenatally screened in further prevalence models. Third, ideally we would also have information on country of birth—which is a better indicator of HBV risk and might provide a better indicator of HBV prevalence within the antenatal sample and multipliers from the non‐antenatal sample. This information could be used to test whether there were any systematic differences in ethnic background in people tested through antenatal programmes and non‐antenatal settings. Unfortunately, current record linkages cannot provide country of birth yet for all tests. Fourth, we assume that age and gender ratios within the non‐antenatal samples are unbiased—which also requires additional evidence to test and justify this assumption. Finally, the method also assumes that the numerators and denominators match, that is, that ethnic group reported to census is equivalent to reporting of ethnic group to hospitals—which also requires additional evidence to test this assumption.

### 4.4 Implications

Our estimates confirm that overall England has a low endemic prevalence of chronic HBV infection, though the number of people in England with chronic HBV is still relatively common and may be larger than the number of people with HIV or HCV [27, 28]. The higher prevalence of HBV in people from ethnic groups other than White British also indicate unmet need and associated equity issues. Compared to UK‐born populations migrants experience a disproportionate burden of disease, with poorer access and outcomes to healthcare [29, 30, 31]. Barriers for migrants accessing testing and treatment include language, stigma, sociocultural factors and limited understanding of HBV infection and its harms [32, 33].

Due to the asymptomatic nature of hepatitis B infection, individuals can remain undiagnosed for many years until they develop advanced liver disease. Early exploration of estimating the diagnosed proportion using laboratory‐confirmed hepatitis B diagnoses and other established health care datasets, suggest that 38% living with chronic hepatitis B in England were diagnosed, 36% for London and 40% outside London [34]. These estimates and the finding that, within the first year of the BBV opt‐out testing in ED programme, hepatitis B had the highest number of new diagnoses identified [24] supports the understanding that there are large numbers of people with undiagnosed HBV. Early diagnosis should ensure access to care and treatment, and better outcomes. Extensive work has already been undertaken to increase testing coverage for hepatitis C, with novel case‐finding interventions developed and implemented in the community [27]. Case finding approaches in primary care for both HCV and HBV has been shown to be effective and cost‐effective but not yet fully implemented [35]. Offer of BBV testing in a range of settings is a key part of elimination efforts [6].

Additional evidence is needed to test some of the assumptions of the method and validate our estimates. However, our England HBV prevalence estimates are crucial in providing a baseline denominator from which to measure the coverage of HBV diagnosis and care, monitor HBV elimination targets and evaluate the impact of prevention and control strategies on hepatitis B burden.

## Author Contributions

R.S. undertook the analysis and had access to the complete data set; S.M., M.E.R. and M.H. came up with the initial concept. R.H., A.G.L. and D.L. provided input on data analysis methods. All authors provided critical input to the manuscript and approved all revisions.

## Conflicts of Interest

The UK Health Security Agency Public Health Programmes Directorate (including immunisation and hepatitis teams) has received money from vaccine and medicines manufacturers on a cost recovery basis for production of surveillance reports on some vaccine preventable diseases to help with manufacturers post licensing requirements. MH received travel support for attending VPHB Antwerp 2024.

## Data Availability

The data that support the findings of this study are available from the corresponding author upon reasonable request.
